# EXPLORING CAREGIVING NETWORK CHARACTERISTICS FOR OLDER ADULTS LIVING WITH DEMENTIA ACROSS RACE/ETHNICITY

**DOI:** 10.1093/geroni/igae098.0591

**Published:** 2024-12-31

**Authors:** Wen-Hua Lai, Natasha Nemmers, Sophia Tsuker, Amanda Leggett

**Affiliations:** Wayne State University, Detroit, Michigan, United States; Wayne State University, Detroit, Michigan, United States; Wayne State University, Detroit, Michigan, United States; Wayne State University, Detroit, Michigan, United States

## Abstract

Black and Latinx older adults face a heightened risk of dementia or cognitive impairment. While minority communities often have family-focused supportive networks compared to White individuals, limited research has explored racial/ethnic disparities in caregiving networks (CGN) and contexts for people living with dementia (PLwD). This study investigates whether CGNs and caregiving contexts vary across racial/ethnic groups. Data from the 2015 National Health and Aging Trends Study (N=2,795) were used to analyze a subgroup of Black, Latinx, and White PLwD. Latent profile analysis identified dementia CGN profiles for each racial/ethnic group based on CGN size and composition (e.g., spouse, children, extended kin, non-kin). Multinomial logistic regression examined caregiving contexts associated with these CGN profiles for each race/ethnicity. Four CGN profiles were identified for each racial/ethnic group. The majority of Black PLwD (80%) were categorized into the “children and others (kin and non-kin)” group, with less spousal involvement. Latinx PLwD had smaller CGN profiles but a more balanced caregiver combination than Black peers. While some similarities in CGN configuration were observed across racial/ethnic groups, compositional differences were evident, influenced partly by sociodemographic and cultural contexts. These findings emphasize the importance of considering CGN disparities by race/ethnicity. Understanding these differences can inform the development of screening and intervention strategies to support caregivers within Black and Latinx families, who disproportionately bear caregiving responsibilities for PLwD.

